# Supplementary material for: Identification of photoperiod-induced specific miRNAs in the adrenal glands of Sunite sheep (Ovis aries)
Source: Front Vet Sci. 2022 Jul 22;9:888207. doi: 10.3389/fvets.2022.888207 (PMC9354845; doi:10.3389/fvets.2022.888207)
Supplement: Supplementary file 1 [file Data_Sheet_1.ZIP › Supplementary file/miRDeep2 file.zip!/miRDeep2 file/Know_Structure/LP42A3/oar-mir-487b.pdf]

miRBase precursor : oar-mir-487b
Total read count : 6044
oar-miR-487b-5p read count : 124
oar-miR-487b-3p read count : 5914
remaining reads : 6

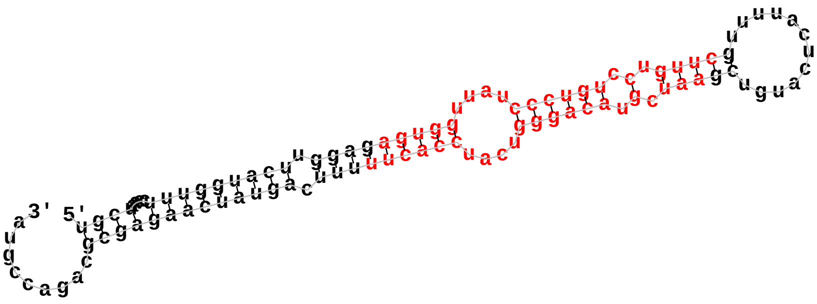

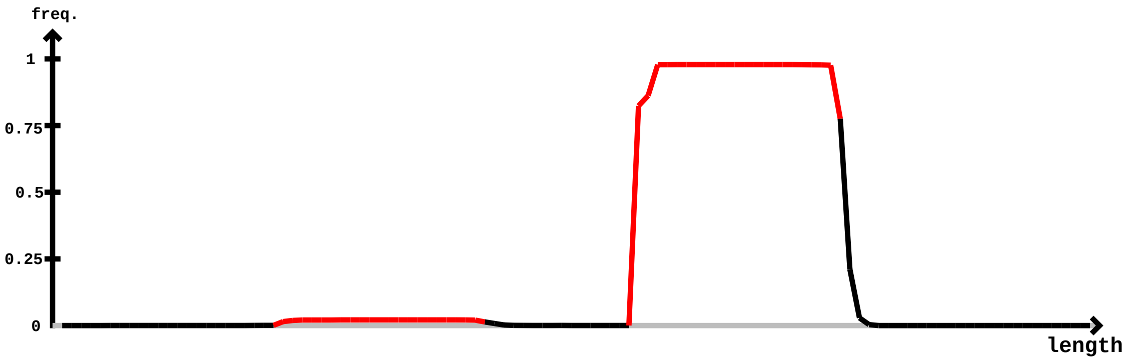


```
                                                                oar-miR-487b-3p
                          oar-miR-487b-5p
5'- ugcuaaccuuugguacuuggagagugguuaucccuguccuguucguuuuacucaugucgaaucguacagggucauccacuuuucaguaucaagagcgcagaccgua  -3'  exp
    .(((....(((((((((.(((((((((....((((((.(.(((((............))))).).))))))....))))))))).)))))))))))))..........  reads  mm  sample
    ....aaccuuugguacuuggaga....................................................................................  1  0  seq
    ...................gagagugguuaucccuguccuguu................................................................  2  0  seq
    ....................agagugguuaucccuguccuguu................................................................  1  0  seq
    ......................agugguuaucccuguccug..................................................................  1  0  seq
    ......................agugguuaucccuguccugu.................................................................  4  0  seq
    ......................agugguuaucccuguccuguu................................................................  36  0  seq
    ......................agugguuaucccuguccuguuc...............................................................  24  0  seq
    ......................agugguuaucccuguccuguucg..............................................................  16  0  seq
    ......................agugguuaucccuguccuguucgu.............................................................  4  0  seq
    ......................agugguuaucccuguccuguucguuuuacu.......................................................  1  0  seq
    .......................gugguuaucccuguccuguu................................................................  2  0  seq
    .......................gugguuaucccuguccuguuc...............................................................  8  0  seq
    .......................gugguuaucccuguccuguucg..............................................................  13  0  seq
    .......................gugguuaucccuguccuguucgu.............................................................  4  0  seq
    ........................ugguuaucccuguccuguuc...............................................................  3  0  seq
    ........................ugguuaucccuguccuguucg..............................................................  5  0  seq
    ........................ugguuaucccuguccuguucgu.............................................................  1  0  seq
    ........................ugguuaucccuguccuguucguu............................................................  1  0  seq
    ............................uaucccuguccuguucguuu...........................................................  1  0  seq
    ............................uaucccuguccuguucguuuuacu.......................................................  1  0  seq
    ...................................................ucaugucgaaucguacagggucauccacu...........................  1  0  seq
    ...........................................................aaucguacagggucauU...............................  1  1  seq
    ...........................................................aaucguacagggucaucc..............................  3  0  seq
    ...........................................................aaucguacagggucaucca.............................  2  0  seq
    ...........................................................aaucguacagggucauccac............................  8  0  seq
    ...........................................................aaucguacagggucauccacu...........................  1175  0  seq
    ...........................................................aaucguacagggucauccacuu..........................  3287  0  seq
    ...........................................................aaucguacagggucauccacuuu.........................  498  0  seq
    ...........................................................aaucguacagggucauccacuuuu........................  3  0  seq
    ............................................................aucguacagggucauccac............................  1  0  seq
    ............................................................aucguacagggucauccacu...........................  31  0  seq
    ............................................................aucguacagggucauccacuu..........................  77  0  seq
    ............................................................aucguacagggucauccacuuu.........................  109  0  seq
    ............................................................aucguacagggucauccacuuuu........................  15  0  seq
```

```
                                                           oar-miR-487b-3p
                      oar-miR-487b-5p
ugcuaaccuuugguacuuggagagugguuaucccuguccuguucguuuuacucaugucgaaucguacagggucauccacuuuuucaguaucaagagcgcagaccgua

............................................................aucguacagggucauccacuuuuu.......................    1    0    seq
.............................................................ucguacagggucauccacu...........................    7    0    seq
.............................................................ucguacagggucauccacuu..........................    44   0    seq
.............................................................ucguacagggucauccacuuu.........................    496  0    seq
.............................................................ucguacagggucauccacuuuu........................    135  0    seq
.............................................................ucguacagggucauccacuuuuu.......................    20   0    seq
...............................................................guacagggucauccacuu..........................    1    0    seq
```
